# Rethinking Amino Acid Nutrition of Black Soldier Fly Larvae (*Hermetia illucens*) Based on Insights from an Amino Acid Reduction Trial

**DOI:** 10.3390/insects15110862

**Published:** 2024-11-04

**Authors:** Andreas Lemme, Patrick Klüber

**Affiliations:** 1 Evonik Operations GmbH, 63457 Hanau, Germany; 2 Fraunhofer Institute for Molecular Biology and Applied Ecology, Department of Bioresources, 35392 Giessen, Germany; patrick.klueber@ime.fraunhofer.de

**Keywords:** black soldier fly larvae, amino acid nutrition, methionine, nutrient modelling, microbial activity

## Abstract

Rearing black soldier fly larvae (BSFL) in order to utilize biogenic materials is of increasing interest in the context of sustainable animal production. However, little is known about the amino acid (AA) requirements of this animal species. Therefore, a feeding experiment with BSFL was conducted, in which lysine, methionine, cysteine, arginine, phenylalanine, or histidine was reduced by 65% or methionine was increased by 65%. Reductions in single AAs in the substrate did not negatively impact BSFL growth, while the addition of methionine improved the growth performance, indicating that methionine was a limiting nutritional factor for maximal productivity. Differences in dietary AA profiles had no impact on the AA profile of the BSFL biomass. However, balance calculations for individual AAs indicated that the substrate microbiome was capable of synthesizing and balancing those AAs for microbial protein synthesis. While both the BSFL and microbiome utilized all free AAs, suggesting that supplemental AAs can be effective additives in BSFL production, this microbial AA synthesis avoided the negative performance impacts of BSFL provoked by severe AA reductions. The quantification of these effects suggested that the microbiome could add up to 35% to the overall AA supply in order to overcome AA deficiency. This effect may not necessarily ensure maximal BSFL productivity, as demonstrated by the extra addition of methionine to the substrate. Our research indicated that BSFL nutrition should consider interactions between substrate composition and microbial activity.

## Simple Summary

While insect larvae production is established in the context of sustainability, meaning that larvae can utilize biogenic by-products from feed and food processing, it also turns out that in insect production, feed costs contribute considerably to overall production costs. With respect to black soldier fly larvae (BSFL) production, the main end product is protein meal, which is used as an ingredient in animal nutrition. By definition, optimizing the protein meal yield requires an understanding of the amino acid requirements of BSFL in order to balance the substrate for efficient utilization. The research reported in this article approached this topic with an amino acid reduction method, which usually allows for determining the ideal ratios between amino acids for optimal performance. Our study revealed that substantial reductions in single amino acids in substrates for BSFL did not impair the performance. However, balance calculations on the fate of dietary amino acids indicated considerable additions of these reduced amino acids by the microbiome. By quantifying these effects, we showed that these additions could be as high as 35% compared to the optimal dietary amino acid supply.

## 1. Introduction

The production of black soldier fly larvae (*Hermetia illucens*, BSFL) has been established globally in recent years. Advancements in production and processing technology have facilitated the large-scale rearing of BSFL, which can be attributed to the insect’s affinity to thrive in a wide array of organic substances [1]. These can include by-products from grain, oil seed and food processing, food waste and by-products from animal processing, and even manure from, e.g., animal production systems [2,3,4,5]. The literature regarding growth studies on different types of substrate indicates large performance differences in BSFL, which can be traced back to impacts on survival, the speed of physiological development, final body weight and biomass, protein and fat deposition, and the efficiency of substrate conversion [2,3,4,5]. However, while all these key production indicators depend largely on the production settings, such as the temperature regime, the moisture content of the substrate, the structure and physical characteristics of the substrate, BSFL dosing per kg, the thickness of the substrate layer, and many others, supplying the larvae with essential nutrients and energy also determines the growth response [1]. While BSFL should have, like any other farm animal species, requirements for nutrients and energy for their optimal growth and health, only little is known about their amino acid (AA) requirements [6]. Koethe et al. [7] increased dietary lysine by 0.5, 1.0, and 3.0% in BSFL diets and observed a significant linear decrease in survival, whereas their body weights did not differ significantly. Some research was conducted to look into the effects of AA in adult insects and their effects on reproduction, but—to the knowledge of the authors—there was no investigation into their effects on weight development and the productivity of larvae. The addition of 31 amino compounds was evaluated in terms of their potential effects on cold tolerance in various life stages of *Drosophila melanogaster* [8]. For arginine and proline, the highest potential to stimulate cold tolerance was described, although other amino acids (asparagine, glutamine, branched-chain amino acids, threonine) also had effects compared to the control. While methionine is an essential amino acid in insects [9], it has also been proposed as an effective larvicide against mosquitoes, although methionine concentrations of <0.25% did not have a negative impact on survival [10]. Interestingly, 3% dietary lysine also significantly reduced the survival of BSFL compared to lower dietary lysine levels [7]. More recently, the effects of AA supplementation in growing larvae have been presented in conferences, but they have not yet been published in peer-reviewed journals. Accordingly, BSFL growth responded to additions of individual essential AAs, but the results do not allow for proposing an optimal amino acid composition in substrates for BSFL production [11].

An approach to determining an “ideal” protein feed was applied in broiler chickens and laying hens [12,13,14]. Single AAs were substantially reduced in a semi-purified experimental diet, and the magnitude of the negative performance response compared to a control diet was used to determine an optimal AA feed composition for these poultry species [12,13,14]. Davis [9] reduced all 20 AAs individually in experimental diets for yellow mealworm (*Tenebrio molitor*). Accordingly, yellow mealworm required the same ten essential amino acids as vertebrates. Moreover, AAs, such as alanine, cysteine, proline, and aspartic acid, appeared conditionally essential. More recently, ten essential AAs were individually reduced in a semi-purified diet for Mediterranean fruit fly (*Ceratitis capitata*) based on corncob [15,16]. While reductions in single AAs had significant impacts on growth, development, and fecundity, no optimal dietary AA pattern could be derived.

Therefore, like Chang [15] with Mediterranean fruit fly or others with broilers and laying hens [12,13,14], an AA reduction experiment was conducted with BSFL to investigate the effects of considerable reductions in single AAs in the substrate on various performance criteria in order to understand the order of the performance limitation of the AAs under examination. It was assumed that sulfur-containing AAs would play an important role in BSFL. For example, it was reported that the methionine concentration in BSFL increased linearly until 30 days of age, while this was not the case for cysteine [17]. Others reported the strongest response on growth by methionine compared to other AAs [11]. Therefore, methionine and cysteine were not only reduced in the current trial but methionine was increased in another treatment. In addition, lysine, arginine, histidine and phenylalanine were reduced. 

## 2. Materials and Methods

The feeding experiment with BSFL was conducted at the Fraunhofer Institute for Molecular Biology and Applied Ecology, Giessen, Germany.

### 2.1. Insect Rearing

BSFs were obtained from Bio.S Biogas (Grimma, Germany) in July 2018. Adult flies were kept in 60 cm × 60 cm × 90 cm (l × w × h) mesh cages (Bioform, Nuremberg, Germany) located in a greenhouse at 26 ± 1 °C, 60 ± 5% relative humidity, and a 12 h photoperiod. Water was provided ad libitum using water-soaked paper towels. Additionally, the cages were sprayed daily with water. Egg clutches were collected daily from wooden egg traps and weighed with an ALJ 160-4A balance (Kern & Sohn, Balingen, Germany). Then, 150 mg (~6000 eggs) was carefully transferred in 19.5 cm × 16.5 cm × 9.5 cm (l × w × h) plastic boxes and sprayed with water. As soon as ≥50% of the eggs had hatched, larvae were fed with 10 g of ground chicken feed (GoldDott Eierglück, DERBY Spezialfutter, Münster, Germany). The moisture content of the feed was adjusted to 70% (TMT-MC-7828S soil moisture meter, OCS.tec, Neuching, Germany) by spraying. The larvae were reared in a climate chamber at 27 ± 1 °C and 65 ± 5% relative humidity in darkness. Once the larvae reached a manageable size of 3–4 mm (instar L3), they were used for subsequent feeding trials.

### 2.2. Experimental Diets

Experimental feeds were based on a prior experiment, in which the effect of starch and AA additions to depectinised apple pomace (DAP) was investigated [18]. The diets, as shown in Table 1, were based on DAP and corn starch. The apple pomace product contained 8% crude protein (CP), 4% ether extract, 30% crude fiber and 10% ash in DM. Corn starch and a mixture of crystalline AA were added to DAP to mimic an AA profile typical for commercial laying hen feed. While semi-purified diets using AA mixtures and achieving CP of about 10% were used for mealworms and Mediterranean fruit flies [9,15,16], the CP content of the diets was 12.2% in DM on average. This level and the AA profile were sufficient to optimize BSFL performance in a previous trial in which graded dietary CP levels from 9.7 to 19.3% in diets based on apple pomace were tested [19]. The current experiment comprised eight treatments, including a positive control (PC), six diets in which single AAs were reduced by about 65% (methionine (-Met), cysteine (-Cys), lysine (-Lys), arginine (-Arg), phenylalanine (-Phe), histidine (-His), and a treatment in which methionine was increased by 65% (+Met). While the AA mixture accounted for 7.50% of the entire composition in PC, the proportion was slightly reduced in the other diets because of reduction and addition of single AAs and varied between 7.06 and 7.37%, except for +Met, where it was 7.63%. Moreover, while 56.6 to 57.2% DAP in the diets accounted for 4.5 to 4.6% CP, the AA mixture contributed about 63% of overall nitrogen (N). All diets were analysed for CP (N × 6.25), total AA, and free AA [20,21,22], confirming intended concentrations. Samples of BSFL biomass as well as of frass were analysed for N, total AA, free AA and ether extract [20,21,22]. The difference between the analytical procedure of total AA, which recovers AA from protein after hydrolysis, and unbound AA to the analytical procedure for free AA is a preceding extraction process. With cold hydrochloric acid, free supplemental AAs as well as free AAs from feed ingredients that are usually present in very little amounts are extracted for analysis [23].

### 2.3. Feeding Trial and Data Collection

First, the moisture content of each diet was determined thermogravimetrically (M35 Moisture Analyzer, Sartorius, Göttingen, Germany) in triplicate in order to calculate the weight of 25 g DM. Subsequently, the respective amount of feed was weighed into 17.0 cm × 8.5 cm × 6.5 cm polypropylene boxes (l × w × h). Tap water was added until a moisture content of 70% was achieved. Each box was stocked with 100 pre-fed juvenile larvae. These were collected from the CF substrate using spring steel tweezers, cleaned, counted, and weighed. Based on this, the initial larval weight was determined and documented. The eight diets were examined as biological triplicates. All approaches were incubated in a climate chamber (27 ± 1 °C, 65 ± 5% relative humidity, 24 h darkness). No additional feed was provided throughout the experiment. The growth of the larvae was recorded at 4-day intervals with a sample size of *n* = 25. The larvae were randomly picked from the substrate using spring steel tweezers, cleaned of coarse impurities, and their weight determined with an analytical balance (AT261 DeltaRange, Mettler, Gießen, Germany). As soon as ≥50% of the individuals within a replicate reached the prepupal stage, the corresponding box was harvested. Here, the larvae were cleaned, counted, weighed, and frozen until further use. The remaining substrate (frass) was also weighed and stored at −20 °C. Since the chemical analysis and performance parameters refer to the DM, larvae and frass were homogenized under liquid N and then lyophilized for 72 h. Prior to this, the moisture content was determined.

The period from the start to the end of feeding was defined as the larval development time. The initial larval weight introduced was subtracted from the harvested wet biomass of the larvae at the end of development. As the larvae were pre-fed, their initial weight was subtracted from the harvested fresh biomass at the end of development. The ratio between the number of introduced (*n* = 100) and collected larvae at the time of harvest was defined as the survival rate. The final individual weight of larvae was calculated by dividing the harvested fresh biomass by the number of surviving larvae.

### 2.4. Data Processing and Statistics

The following performance indicators were calculated and statistically analysed:

Equation (1)—gain:

(1) $$A,\frac{g}{replicate}=B,\frac{g}{replicate}-C,\frac{g}{replicate}$$

with: *A* = BSFL biomass gain (fresh or dry matter), fat gain, crude protein gain, or amino acid gain in g per replicate;

*B* = BSFL biomass at termination of experiment (fresh, dry matter), biomass at termination of experiment × ether extract, crude protein, or amino acids in g per replicate;

*C* = BSFL biomass at start of experiment (fresh, dry matter), biomass at start of experiment x ether extract, crude protein, or amino acids in g per replicate.

Equation (2)—conversion efficiency:

(2) $$A,\frac{g}{g}=\frac{B,\frac{g}{replicate}}{C,\frac{g}{replicate}-D,\frac{g}{replicate}}$$

with:

*A* = conversion efficiency in g/g;

*B* = BSFL biomass at termination of experiment in g dry matter;

*C* = Substrate at start of experiment in g dry matter;

*D* = Frass at termination of experiment in g dry matter.

Equation (3)—substrate conversion:

(3) $$A,\frac{g}{g}=\frac{B,\frac{g}{replicate}}{C,\frac{g}{replicate}}$$

with:

*A* = substrate conversion in g/g;

*B* = substrate at start of experiment in g dry matter;

*C* = BSFL biomass gain in g dry matter.

Crude protein (nitrogen × 6.25), individual AA and sum of AA were quantified in substrate, final BSFL biomass and frass per replicate on a DM basis. The data were used for calculations of losses according to the following formula:

Equation (4)—loss:

(4) A,g=B,g−(C,g+D,g)

with:

*A* = loss of nitrogen or amino acid in g per replicate;

*B* = nitrogen or amino acid in substrate in g per replicate at start;

*C* = nitrogen or amino acid in BSFL biomass in g per replicate at termination of experiment;

*D* = nitrogen or amino acid in frass in g per replicate at termination of experiment.

For comparison reasons, amounts of BSFL biomass, frass, and loss were expressed in % of substrate.

## 3. Results and Discussion

Substrates were analysed for crude nutrients and AAs. The calculated nutrient levels shown in Table 1 were confirmed.

Performance data of BSFL raised on diets differing in their AA supply are shown in Table 2. Essentially, individual weights increased until day 12 and declined considerably thereafter. The growth curve was flattest for the positive control, and body weights at day 12 were significantly lower than those for -Cys and +Met treatments (*p* < 0.05), while body weights in -Arg, -Phe, and -His were in between. Body weights recorded for -Met and -Lys were almost identical to PC. Indeed, individual body weights declined until day 16, but the weights of PC were still lowest, while they were highest for -Phe (*p* < 0.05). All other treatments were in between and did not significantly differ from PC or -Phe. As there were differences in survival, the highest biomass gain was achieved with +Met, while PC produced the lowest amount of biomass (*p* < 0.05). However, while the biomass of -Arg, -Cys, -His, and -Lys was only numerically but not significantly lower than that of +Met, the biomass of -Phe, and -Met differed significantly (*p* < 0.05). 

The response of BSFL growth to different diets was unexpected as the concept of a deletion approach would suggest that a reduction in single AAs reduces growth because important building blocks for protein synthesis are missing. When single AAs were reduced in diets for meal worm, the gain was considerably reduced, particularly with a reduction in lysine, methionine, threonine, arginine, valine, isoleucine, leucine, tryptophan, histidine, and phenylalanine that were, thus, considered essential [9]. In contrast, the impact of glycine, serine, alanine, cysteine, proline, glutamic acid and aspartic acid reduction was marginal compared to the positive control [9]. An individual reduction in lysine, methionine, valine, isoleucine, leucine, arginine, histidine and phenylalanine in the substrate for Mediterranean fruit fly larvae reduced growth to less than 50%. Reductions in threonine and tryptophan even resulted in complete mortality [9]. Interestingly, a reduction in lysine, for example, was in a similar range compared to our trial. The concept for the AA reduction approach was applied in broilers and laying hens in order to determine an ideal AA profile relative to lysine [12,13,14]. The researchers also prepared control diets, in which a large proportion of the entire dietary AA was provided with free AAs and experimental diets in which single AAs were substantially reduced. As observed in meal worms and fruit flies [9,15], these reductions resulted in depressions in various performance parameters such as N retention, allowing for estimating an ideal dietary AA pattern for broilers and laying hens [12,13,14]. The reduction in methionine, cysteine, lysine, arginine, phenylalanine or histidine in the current BSFL model did not negatively impact growth or BSFL development but rather increased body weights and biomass, although this was not significant in most cases (Table 2). While body weights and biomass of BSFL also include fat deposition and N deposition (expressed as CP, N × 6.25), the deposition of the sum of AAs might be more sensitive. The respective results do not suggest a severe negative impact on these criteria due to the reduction in single AAs (Table 2), which is in contrast to similar experimental setups tested on other insects [9,15]. Indeed, -Phe showed a 12% lower CP and AA deposition than the PC, but this was not significant. Finally, a similar conclusion can be drawn with respect to conversion efficiency and substrate conversion (Table 2).

In contrast, it appeared that the addition of methionine to the substrate was beneficial as body weights at days 8 and 12 were higher than the positive control (*p* < 0.05; Table 2). However, they were only numerically higher compared to some AAs, for example, -Phe or -Arg. The biomass was also maximized with +Met and significantly higher than PC, -Met, and -Phe (*p* < 0.05). When expressed on DM, this advantage disappeared, and the biomass of +Met did not differ from either treatment. Interestingly, the fat gain of +Met BSFL was significantly lower than PC as well as—Cys (*p* < 0.05), whereas N and AA gain was no different from all other treatments. When substrates were supplemented with graded levels of lysine, increasing CP content and a trend of declining fat content in BSFL were reported, while the overall growth was not affected *[7]*. The lower fat deposition with higher AA supply may indicate a more efficient protein deposition at the expense of energy storage in the form of fat. However, increased protein gain was not observed in treatment +Met, although the growth pattern indicated that the addition of methionine had a strong impact because weights at day 12 were ~24% higher than those achieved with PC. This could be because the ideal harvest day would be earlier, as a weight reduction after day 12 equalized the biomass of all treatments to a certain extent. Indeed, ≥ 50% of the individuals within a replicate already reached the prepupal stage, which implies that these individuals no longer consumed feed and were, thus, mobilizing energy and, therefore, losing weight [24].

The growth rate and final performance of the BSFL appear low. However, the experimental diets were based on depectinised apple pomace. Broeckx et al. [2] reported the flattest growth curve and the lowest bioconversion efficiency for a similar by-product from apple processing (apple pulp) used in a trial testing 14 different materials as substrates. We could demonstrate that the addition of starch or an AA mixture or both to the DAP can considerably improve the growth and biomass yield [18]. Moreover, based on this DAP–starch–AA mixture concept, 12.2% CP was found to be sufficient [19]. A CP level of about 12% was sufficient for optimizing BSFL growth, as reported by Schneider et al. [25]. Recent modelling efforts also suggested that 12% CP could be sufficient for BSFL growth, provided sufficient dietary lipids of >10% are in the substrate. Indeed, a fat content of 2.6% in the current trial (Table 1) is not in line with that publication. However, only the addition of methionine seemed to improve the performance of BSFL compared to PC, suggesting that methionine limited the growth of the larvae. When single AAs were added to the Gainesville diet, doubling the methionine content resulted in the strongest response both on weight gain and the feed conversion ratio [11]. However, the addition of other AAs to the Gainesville diet also increased body weights and improved the feed conversion ratio significantly [11], which questions the applicability of the ideal AA concept for BSFL growth. In addition, the absence of negative responses in the deletion approach supports this conclusion.

The biomass gain can be split into fat gain and CP or AA gain (Table 2). Interestingly, the sum of CP and fat gain is well below the DM biomass gain. It can be speculated as to whether the factor 6.25 (assuming 16% N in protein) to calculated CP from N is correct. On average, across all treatments, 7.095% N was found in BSFL biomass DM. Only 4.881% N was provided by AAs, accounting for 33.94% of the diet, suggesting 14.4% N or an appropriate factor of 6.95. About 2.214% N was provided by other compounds, possibly chitin, to a large extent. Indeed, chitin was not analysed in the current study, but it contains 6.9% N [26], equivalent to a factor of 14.5%. Consequently, N would explain 66.0% of DM (4.881 × 6.95 + 2.214 × 14.50) instead of 44.3% (7.095 × 6.25). Accordingly, 20% of DM is unexplained, and CP is underestimated by 37% if it is calculated conventionally. It might be added that other non-protein N compounds can be found in biogenic materials (e.g., nucleic acids, nucleotides, vitamins, urea, etc.), which would further impact the overall factor and weight proportion. However, the above equation suggests a weighted conversion factor of 9.31 ((4.881 × 6.95 + 2.214 × 14.50)/(4.881 + 2.214)), which differs considerably from findings by others reporting a conversion factor of 4.67 for BSFL, a value which is even lower than 6.25 [27]. Adjusting the CP gain in Table 2 accordingly would result in an average CP gain of 1.71g/rep. Together with a 0.46 g/rep fat gain, this would add up to 2.17g/rep and, thus, the gap to DM BSFL biomass gain would shrink to 0.42 g/rep. Still, this accounts for 16% of overall DM gain, and the explanation for this remains unclear, although carbohydrates in the remaining gut fill of BSFL might explain at least part of it. Despite these findings, significant differences in protein gain and fat gain were found between treatments (Table 2; *p* < 0.05). Earlier research by our group indicated that, particularly, the addition of starch to DAP allowed for fat deposition, while the addition of an AA mixture to DAP allowed for preliminary protein deposition [18]. Others reported that rather low dietary protein of about 10% in combination with 35% dietary carbohydrates maximized the larval CP content, while the larval crude fat content was maximized with dietary protein levels of 24%, and dietary carbohydrates did not have a big effect [28]. However, the maximal larval yield was achieved with high dietary protein (>22%) and high carbohydrates (>50%), and it is not clear how these parameters affect the larval protein and fat yields [28]. Apart from such studies focusing on the impact of crude nutrients, no research examining the effects of single AAs on BSFL composition could be found. Growth responses were already unexpected (see above), as were the effects on protein and fat yield.

### 3.1. Nutrient Balance Model

The analyses of substrate, BSFL biomass, and frass allowed for nutrient balancing. The balance for N and AA expressed in % of the substrate is presented in Table 3. Accordingly, between 33.1 and 42.0% of the N supplied with the substrate was captured in the biomass of BSFL. Nitrogen retention was significantly higher in -Phe than in -His (*p* < 0.05), whereas all other treatments did not differ statistically. Confirming the protein deposition responses (Table 2), neither the reduction in single AAs nor the addition of methionine impacted the N retention efficiency. Moreover, the retention efficiency of AAs (sum of AAs) did not reveal any significant treatment differences. However, it is remarkable that the AA retention efficiency was 20–25% lower than N retention (8 and 11%-points), indicating that N was deposited in the form of other N-containing constituents than AAs (see above). According to Soetemans et al. [29], BSFL biomass contains 7.8–9.5% chitin, which would explain about 3.4–4.1% CP. The average CP content of BSFL biomass in our study was 44.3% DM across all treatments (42.8% min; 46.7% max), from which only 69% was explained by AA–nitrogen. This appears to be much higher than reported by Soetemans et al. [29], and it might be speculated whether other N-containing compounds can be found in BSFL or whether substantially higher chitin levels are present in BSFL at harvest or both. These retention efficiency results suggest that about 70% of the AAs provided with the feed could still not be converted into biomass, which is of economic importance.

Between 45 and 58% of dietary N was found in frass, while the sum of AA levels ranged between 34 and 42%. The highest relative concentrations were found in Phe, while the lowest were in -His (*p* < 0.05). The difference between the N and AA ratio to substrate might be, again, explained by the high chitin load in frass, although the frass was not explicitly analysed for chitin. Chitin is the main structural constituent of the exoskeleton and other matrices in insects and their larvae [30,31]. While chitin is a polymer, glutamine is required as a N donor for chitin synthesis. Chitin contains 6.9% N, being equivalent to 43.1% CP. Larvae biomass contains 7.8–9.5% chitin, which increases to 10.3–10.7% in pupae, but shedding and shells, which will be found in frass after harvest, would contain even up to 31.1% chitin [29]. The latter explains the high N proportion in frass as 31.1% chitin in shedding would be equivalent to 2.1% N. Interestingly, the average N content (in DM) across all treatments was 2.3% (2.0% min; 2.5% max), which, indeed, was not 100% chitin, as 1.4% N was explained by AAs on average. That would mean that about 0.9% N in frass can be attributed to chitin, accounting for 40% of overall N.

The difference between the sum of biomass- and frass-N to substrate-N quantities is defined as loss. Nitrogen losses ranged between 5% (-Lys) and 15% (+Met; *p* < 0.05). This seems to be in line with N emissions of about 7.5% (relative to substrate) reported for BSFL raised on brewers spent grain diets with 13.8% CP in DM [32]. When a diet with 43.4% CP based on supermarket waste was used, N losses were 15.2% of the substrate [32]. While losses in our study cover a similar range to those reported by Coudron et al. [32], they appear relatively low compared to DAP-based diets with higher CP levels [19], where the N losses were as high as 31% at 19.3% dietary CP. Lalander et al. [33] used a substrate with about 37% CP and reported N losses of about 44%. Nitrogen losses can be assigned to ammonia losses to a large degree [32,33]. N losses in the form of ammonia have a close relationship with microbial activity in the substrate. A strong increase in ammonia concentrations in various substrates for BSFL has been associated with the substrate microbiome, which itself was manipulated by substrates [34]. Earlier research associated ammonia production to the BSFL themselves when comparing to controls without BSFL; however, in that case, the inoculation of the substrate with the BSFL-related microbiome was avoided [35]. Such an inoculation would be beneficial for optimal substrate utilization [36]. The range of N losses found in the current experiment covers factor 3 (5% with -Lys to 15% with +Met), and it remains unclear whether a reduction or increase in single AAs can have a strong impact on N conversion by the microbiome. This would suggest that additional methionine stimulates microbial N conversion to a higher degree than a substantial reduction in lysine, despite the similar deposition.

While N losses can be attributed to ammonia losses [32,33], AA losses cannot (Table 3). First, AA losses were substantially higher than N losses and always greater than 30%. The fate of dietary AAs can be different as they can be incorporated into BSFL biomass, incorporated into microbial biomass, used as precursors for other constituents in the metabolism of both BSFL and microbiome, or degraded and used energetically. Particularly, the last two possibilities will explain AA losses in the production system, although it is not possible to distinguish between the effects of BSFL and microbiome with the current experimental approach. However, it is remarkable that, while about 63% of total N in the experimental diets was provided in the form of free, crystalline AAs, only protein-bound AAs, rather than free AAs, could be detected in frass in all treatments after the termination of the experiments. It is, therefore, concluded that the entire amount of free AAs was consumed and utilized by BSFL and the microbiome. Consequently, the protein of frass consisted only of non-consumed DAP protein and microbial protein. It is further concluded that BSFL are well able to ingest and use free AAs, which offers the opportunity to use them as feed additives to optimize the AA supply of BSFL. The above-discussed effects of treatment +Met already provide evidence for this. It remains unclear why the sum of AA losses differed between treatments, but there were similarities to N losses, and it might be that considerable changes in the dietary AA profile affected the composition of the microbial community and, thus, the utilization of the substrate, as observed in BSFL growth on chicken feed or cottonseed press cake [34,37]. Overall, the data show that more than two-thirds of the dietary AAs were not used for the BSFL biomass yield, and about one-third of dietary AAs were even lost from the system. This is somehow in line with the mass balance calculation by Klammsteiner et al. [34], reporting that only 28% of dry matter was retained in BSFL grown on chicken feed, while 33% was lost from the production system. When BSFL were fed fruit, dry matter losses were as high as 48% [34]. As protein and AAs usually belong to the expensive nutrients in animal production systems, there seems to be a high potential to reduce losses and to increase retention efficiency.

The sum of AAs in BSFL biomass did not significantly differ between treatments but varied within 27–32% (Table 3). Table 4 displays the CP content, the lysine content, and the AA profile relative to lysine analysed for each treatment. The protein content (% DM) ranged from 42.8% (-Phe) to 46.7% (-Met) and the lysine content ranged between 2.018% (-Phe) and 2.318% (-Met). While this appears variable, the lysine to CP ratio was very narrow (4.9–5.1%), except for -Phe with a ratio of 4.7%. Moreover, the AA profile (% of lysine) was very similar between the treatments. Treatment -Arg showed slight deviations as the protein and lysine level in biomass was lowest compared to the other treatments, while, in particular, isoleucine, leucine, valine, serine, alanine and glutamic acid to lysine ratios appeared to be highest. It is questionable whether this is an effect due to arginine reduction in the substrate or whether this is an artefact. It might be expected that the AA profiles of BSFL between treatments are similar as the proteins of tissues are genetically determined. Differences in the AA profile might only occur if there are substantial treatment differences in terms of the growth stage, which would affect the allometric growth of different tissues. A comparison of AA profiles of few life stages would confirm this conclusion [38]. Koethe et al. [7] reported that the lysine content in BSFL dry matter was unchanged with increasing dietary lysine levels and, moreover, the reported results suggest that the lysine concentration within crude protein was also unaffected, which is basically in line with our observation. Miner et al. [17] reported that the cysteine content in BSFL did not change with an increasing dietary carbon/nitrogen ratio, whereas the methionine content increased. However, they also reported that the methionine content increased linearly with age, while this was not the case for cysteine. In addition, it was not clear whether fat deposition was also an influencing factor. Fuso et al. [39] also reported the dietary impact on the AA profile of BSFL. First, they found a lysine to CP ratio between 5.2 and 6.3%, which was, thus, higher and more variable than in our study. Moreover, the AA profile (% of lysine) differed considerably between treatments, although no growth responses and potential differences in life stages were reported. For example, in our case, methionine or cysteine to lysine ratios were consistently about 30% or 12%, while they ranged between 28% and 37% or 29% and 39% in the aforementioned study [39]. The cysteine ratio was, thus, more than double in the current study and is very different when compared to the cysteine/lysine ratios of 13.6 to 15.1% analysed in 83 BSFL meals with protein contents ranging from 30% up to >60% *[40]*. In conclusion, the AA profile of BSFL protein was consistent between the dietary treatments, which is associated with a similar average instar at harvest time. Moreover, manipulation of the AA profile within the same instar is difficult due to the underlying genetic code. However, differences in genetic origin per se as well between instars may explain differences between the literature and the current study.

### 3.2. Amino Acid Retention Efficiency

Balances were calculated not only on N and the sum of AAs (Table 3) but also on individual AAs (Table 5 and Table 6). The retention efficiency (mg retained/mg substrate) of individual AAs is shown in Table 5. Accordingly, there were considerable differences between AAs and also within AAs (between treatments).

With respect to AA retention efficiency (Table 5), the low variation between treatments within AAs is remarkable. For lysine, methionine, cysteine, arginine, phenylalanine, and histidine, there were no differences between treatments, except those AAs which were reduced in -Lys, -Met, -Cys, -Arg, -Phe, and -His. These were significantly higher compared to the other treatments (*p* < 0.05). This reflects a mathematical phenomenon rather than a biological one because the levels of these reduced AAs were extremely low when compared to the respective other treatments. In fact, retention in mg/replicates did not show differences between treatments, e.g., methionine retention in -Met and +Met amounted to 16.9 and 16.7 mg/replicate, while the other sis treatments had on average 16.9 mg/replicate. Lysine retention in -Lys was 56.7 mg/replicate and, in the other treatments, 57.0 mg/replicate. There were also no significant treatment differences in retention efficiency for threonine, leucine, serine, aspartate, and glutamate. Valine, isoleucine histidine, glycine, proline, and alanine were significantly higher in -Cys (and partly -His) compared to -Phe (*p* < 0.05), whereas all other treatments were in between and not different (Table 5). This pattern is similar to the DM gain in BSFL and might be directly associated with general growth effects. Overall, it appears that retention efficiencies between treatments were rather similar except for reduced AAs. This is surprising because a limiting AA would interrupt the translation of the genetic code into protein synthesis and, consequently, reduce the deposition of all AAs and, therefore, impair their retention efficiencies [41]. Indeed, similar retentions in mg/replicate indicate that there was no limitation of any other AA.

Having very similar AA retentions between treatments in mind (mg/replicate, see above), the retention efficiencies of reduced AAs are remarkable. Particularly, lysine, histidine, methionine, and arginine retention efficiencies were 77% (-Arg) up to 94% (-Lys), suggesting that BSFL almost completely captured the AA load offered with the substrate in these cases. In contrast, the methionine retention efficiency was significantly lower in +Met compared to all the other treatments (*p* < 0.05) except for -Met, which was even higher (*p* < 0.05). This seems to confirm the law of diminishing returns, suggesting that each additional unit of offered nutrient is utilized to a lower degree [42].

While the retention efficiencies of non-essential AAs are potentially influenced by transamination and de novo synthesis, this is, by definition, not the case for essential AAs. However, differences were substantial and ranged from 15.6% for cysteine (average of all treatments except -Cys) to 38.5% for lysine or 39.2% for valine. It is difficult to assess whether a ranking between the AA retention efficiency indicates a general order of potential limitation, in that, the lower the supply compared to the requirement, the higher the retention efficiency. For example, for farm animals, it is known that lysine is almost exclusively used as a building block in proteins, while, for example, a high percentage of cysteine or methionine+cysteine are needed as precursors for metabolic processes, such as being a methyl donor (methionine), a precursor of cysteine (methionine), a precursor of glutathione (cysteine), and others [43]. The latter would mean that these AAs are not deposited in the animal, resulting in a lower retention efficiency. For example, Edwards III and Baker, Edwards III et al. [44,45] estimated a retention efficiency of 79% for ingested lysine in broilers, whereas only 52% of ingested methionine+cysteine were deposited as methionine+cysteine. However, their use as functional AAs is essential for maintaining all metabolic processes and allows for all adjustments to any kind of exogenous factors. Therefore, a low retention efficiency, as found for cysteine, does not indicate an oversupply. Glutamic acid and glutamine (which, due to analytical procedures, appear only as glutamic acid) were most abundant and three-fold higher than lysine in substrates. However, the retention was less than double that of lysine, while the retention efficiency was only 15.8% on average. All other non-essential AAs (glycine, serine, proline, alanine, aspartic acid) had considerably higher average retention efficiencies between 29.3 and 38.4% (Table 5). Glutamic acid recovery in frass was only 24% of the substrate, whereas 60% were lost on average (Table 6). Obviously, an extraordinarily large proportion of dietary glutamic acid was transformed or degraded by both BSFL and the microbiome. Glutamine is the AA donating the N to chitin formation *[31]*, which might explain the low retention efficiency and high losses, although glutamine becomes glutamic acid after N donation.

### 3.3. Amino Acid Losses

In principle, the results of the sum of AAs (Table 3) can be split up into individual essential and non-essential AAs. In this context, research on Mediterranean fruit fly larvae indicated lysine, methionine threonine, tryptophan, arginine, valine, isoleucine, leucine, phenylalanine, and histidine to be essential and glycine, serine, cysteine, alanine, proline, aspartic acid, and glutamic acid non-essential *[9]*. For BSFL, the same distinction was assumed. In particular, the AA losses raised our attention. There were considerable differences between losses of individual AAs, as they amounted, on average, to 60% for glutamic acid but only to 6% on average for alanine (Table 6). While these two AAs belong to non-essential AAs, the differences were also high within essential AAs as arginine and cysteine losses were as high as 42% and 47%, but valine losses were only 14% on average.

The calculation of AA losses revealed one remarkable phenomenon as losses for those AAs that were reduced in the experimental diets were negative. Negative losses, by definition, mean that BSFL biomass plus frass contained higher quantities compared to quantities offered with the substrate. In other words, AAs were added to the system. The only explanation for this finding is that the microbiome synthesized these AAs from other N sources than AAs. As mentioned earlier, no free AAs were found in frass, suggesting that BSFL and the microbiome incorporated ingested AAs into the body or microbial protein or degraded them. However, it is not only BSFL that have requirements for AAs in order to allow for complete translation of genetic information into proteins but bacteria and other organisms of the microbiota require AAs for this purpose, too; however, for example, bacteria are able to synthesize AAs from inorganic N sources [46,47], which would allow them to satisfy their needs. Obviously, the capability and capacity for AA synthesis are high, as the losses, given in % of the substrate, were high, although these numbers are misleading at first glance because these particular AAs were reduced by 65% compared to PC. However, if these negative losses are related to the levels offered by the other adequate diets, the microbiome added 24%, 5%, 35%, 18%, 20% and 23% of methionine, cysteine, lysine, arginine, histidine and phenylalanine, respectively. These numbers were equivalent to 13.9, 2.2., 52.2, 31.0, 16.7 and 32.3 mg per replicate. It might be speculated whether the respective addition of these amounts would meet the minimum AA requirement of the microbiota and would, thus, avoid the de novo synthesis of AAs. Can it be assumed that the microbiota synthesizes AAs only to a degree to satisfy its own requirements but not to produce a surplus? Only dietary levels above those “minima” would be available to BSFL. Can this hypothesis be confirmed by balanced data? The treatments -Met, -Cys, and +Met serve as suitable examples, as not only methionine and cysteine reduction but also methionine increase is involved. Moreover, it is assumed that, similar to other animals, methionine can be transformed into cysteine if needed [48].

If the above-quantified AA contributions by the microbiome (mg/replicate) are added to the respective quantities supplied with the substrate in these treatments (mostly from DAP, Table 1), 34.8, 21.2, 56.0 and 112.3 mg/replicate methionine, cysteine, methionine+cysteine, and lysine are calculated. However, if these values were the minimum needed for the microbiome and anything above was available for BSFL, negative performance responses should be observed according to the AA reduction concept [12,13,14] because these numbers were still 33%, 53%, 43%, and 24% below mean levels supplied with PC or treatments without respective AA reductions. Therefore, it is hypothesized that the microbiome synthesized and balanced even higher amounts of single AAs, maybe up to the levels provided with PC. This would be remarkable, and the question arises whether severe reductions of two or more AAs can also be balanced by the microbiome. Moreover, if the microbiome adds possibly more of these AAs than a balance study like this can identify, the high retention efficiencies shown in Table 5 would then be overestimates. In any case, it appears that when there is a deficiency, microorganisms activate a mechanism to synthesize AAs from non-organic N [46,47]. However, while 50% more methionine in PC compared to -Met did not have beneficial effects on any performance criterion, 146% more methionine (as tested in +Met) resulted in clear responses, suggesting that methionine supplies PC with limited BSFL development and growth. While a response to additional methionine could be repeated in research by others to indicate that the addition of different single AAs to the Gainesville diet resulted in significant improvements [11].

## 4. Conclusions

A feeding experiment with black soldier fly larvae (BSFL) revealed that severe reductions in single AAs in a substrate did not negatively impact BSFL growth, while the addition of methionine improved the growth performance, indicating that methionine was a limiting nutritional factor for maximal productivity in the substrates.

The comparison of nitrogen (N) and amino acids (AAs) revealed that crude protein being defined as N multiplied with 6.25 is not an appropriate approach, as interactions with chitin result in a substantial underestimation. It is recommended to refer to N or to use a factor considering a N content of 6.9% for the chitin proportion. Moreover, the interaction with chitin formation by the BSFL results in an overestimation of the nutritional efficiency of a substrate when referring to N retention because AA retention efficiencies were about 20% to 25% lower than the N retention efficiency. Consequently, the losses of AAs (not captured in BSFL biomass nor found in frass) were 150% to 470% higher than the N losses.

Differences in the dietary AA profile had no impact on the AA profile of BSFL biomass. However, balance calculations for individual AAs indicated that the substrate microbiome was capable of synthesizing AAs that were missing for microbial protein synthesis. While both BSFL and the microbiome completely utilized all free AAs, suggesting that supplemental AAs can be effective supplements in BSFL production, this microbial AA synthesis avoided performance depressions in BSFL due to severe AA reductions. Still, this effect may not necessarily ensure maximal BSFL growth and productivity, as demonstrated with the addition of extra methionine to the substrate.

Overall, using the AA reduction approach in order to determine an optimal dietary AA profile for BSFL is confounded by microbial activity and, therefore, not successfully applicable. The observed positive responses with the addition of methionine suggests that dose–response experiments might have higher potential to estimate the AA levels required for optimized BSFL productivity. However, only the AA reduction approach could identify and quantify the capability of microbial AA synthesis.

## Figures and Tables

**Table 1:** Ingredients and calculated nutrient composition of experimental substrates including a positive control (PC) and variants in which single amino acids were reduced or increased.

		PC	-Met	-Cys	-Lys	-Arg	-Phe	-His	+Met
Depectinised apple pomace	%	56.72	56.85	56.89	57.16	57.16	57.09	56.92	56.59
Corn starch	%	32.30	32.30	32.30	32.30	32.30	32.30	32.30	32.30
Limestone	%	1.22	1.22	1.22	1.22	1.22	1.22	1.22	1.22
Dicalcium-phosphate	%	1.50	1.50	1.50	1.50	1.50	1.50	1.50	1.50
Salt	%	0.26	0.26	0.26	0.26	0.26	0.26	0.26	0.26
Premix ^1^	%	0.50	0.50	0.50	0.50	0.50	0.50	0.50	0.50
L-Lysine HCl	%	0.49	0.49	0.49	**0.05**	0.49	0.49	0.49	0.49
DL-Methionine	%	0.14	**0.01**	0.14	0.14	0.14	0.14	0.14	**0.27**
L-Cysteine	%	0.21	0.21	**0.04**	0.21	0.21	0.21	0.21	0.21
L-Threonine	%	0.26	0.26	0.26	0.26	0.26	0.26	0.26	0.26
L-Arginine	%	0.47	0.47	0.47	0.47	**0.03**	0.47	0.47	0.47
L-Tryptophan	%	0.05	0.05	0.05	0.05	0.05	0.05	0.05	0.05
L-Valine	%	0.32	0.32	0.32	0.32	0.32	0.32	0.32	0.32
L-Isoleucine	%	0.28	0.28	0.28	0.28	0.28	0.28	0.28	0.28
L-Leucine	%	0.57	0.57	0.57	0.57	0.57	0.57	0.57	0.57
L-Histidine	%	0.24	0.24	0.24	0.24	0.24	0.24	**0.04**	0.24
L-Phenylalanine	%	0.37	0.37	0.37	0.37	0.37	**0.00**	0.37	0.37
L-Tyrosine	%	0.18	0.18	0.18	0.18	0.18	0.18	0.18	0.18
Glycine	%	0.29	0.29	0.29	0.29	0.29	0.29	0.29	0.29
L-Serine	%	0.39	0.39	0.39	0.39	0.39	0.39	0.39	0.39
L-Proline	%	0.55	0.55	0.55	0.55	0.55	0.55	0.55	0.55
L-Alanine	%	0.33	0.33	0.33	0.33	0.33	0.33	0.33	0.33
L-Asparagine	%	0.67	0.67	0.67	0.67	0.67	0.67	0.67	0.67
L-Glutamine	%	1.70	1.70	1.70	1.70	1.70	1.70	1.70	1.70
Dry matter	%	92.3	92.3	92.3	92.3	92.3	92.3	92.3	92.3
Crude protein	%	12.2	12.2	12.2	12.2	12.2	12.2	12.2	12.2
Crude fiber	%	18.2	18.2	18.2	18.2	18.2	18.2	18.2	18.2
Ether extract	%	2.6	2.6	2.6	2.6	2.6	2.6	2.6	2.6
Crude ash	%	9.6	9.6	9.6	9.6	9.6	9.6	9.6	9.6
Calcium	%	0.9	0.9	0.9	0.9	0.9	0.9	0.9	0.9
Phosphorous	%	0.45	0.45	0.45	0.45	0.45	0.45	0.45	0.45
Lys	%	0.52	0.52	0.52	**0.18**	0.52	0.52	0.52	0.52
Met	%	0.20	**0.07**	0.20	0.20	0.20	0.20	0.20	**0.33**
Cys	%	0.20	0.20	**0.07**	0.20	0.20	0.20	0.20	0.20
Thr	%	0.42	0.42	0.42	0.42	0.42	0.42	0.42	0.42
Trp	%	0.10	0.10	0.10	0.10	0.10	0.10	0.10	0.10
Arg	%	0.67	0.67	0.67	0.67	**0.23**	0.67	0.67	0.67
Ile	%	0.45	0.45	0.45	0.45	0.45	0.45	0.45	0.45
Leu	%	0.88	0.88	0.88	0.88	0.88	0.88	0.88	0.88
Val	%	0.51	0.51	0.51	0.51	0.51	0.51	0.51	0.51
His	%	0.31	0.31	0.31	0.31	0.31	0.31	**0.11**	0.31
Phe	%	0.55	0.55	0.55	0.55	0.55	**0.19**	0.55	0.55
Tyr	%	0.36	0.36	0.36	0.36	0.36	0.36	0.36	0.36
Gly	%	0.50	0.50	0.50	0.50	0.50	0.50	0.50	0.50
Ser	%	0.57	0.57	0.57	0.57	0.57	0.57	0.57	0.57
Pro	%	0.71	0.71	0.71	0.71	0.71	0.71	0.71	0.71
Ala	%	0.52	0.52	0.52	0.52	0.52	0.52	0.52	0.52
Asp	%	1.04	1.04	1.04	1.04	1.04	1.04	1.04	1.04
Glu	%	2.27	2.27	2.27	2.27	2.27	2.27	2.27	2.27

^1^ Premix composition: 88% dry matter, 21.15% calcium, 3,000,000 NE/kg vitamin A, 1,000,000 NE/kg vitamin D3, 16,000 mg/kg vitamin E, 20,000 mg/kg zinc, 9000 mg/kg iron, 22,000 mg/kg manganese, 3000 mg/kg copper, 70 mg/kg selenium, 234 mg/kg iodine.

**Table 2:** Final average body weights of BSFL, overall biomass production, and efficiency of substrate conversion at day 16.

	Trt 1 PC	SD	Trt 2 -Met	SD	Trt 3 -Cys	SD	Trt 4 -Lys	SD	Trt 5 -Arg	SD	Trt 6 -Phe	SD	Trt 7 -His	SD	Trt 8 +Met	SD
Individual body weights (mg/larva; *n* = 25 per replicate)																
Day 0	**8.5 ^a^**		**7.4 ^a^**		**7.1 ^ab^**		**6.7 ^ab^**		**6.3 ^ab^**		**5.4 ^b^**		**5.2 ^b^**		**4.8 ^b^**	
Day 4	**50.0 ^ab^**	0.56	**56.9 ^a^**	4.03	**57.8 ^a^**	3.08	**53.3 ^ab^**	3.56	**59.2 ^a^**	6.08	**40.7 ^b^**	2.55	**42.2 ^a^**	2.61	**55.2 ^ab^**	11.12
Day 8	**116 ^b^**	3.8	**117 ^b^**	8.2	**121 ^b^**	9.0	**130 ^ab^**	9.4	**132 ^ab^**	6.5	**119 ^b^**	4.0	**115 ^b^**	8.0	**153 ^a^**	13.1
Day 12	**131 ^b^**	4.6	**131 ^b^**	10.5	**141 ^a^**	5.7	**134 ^b^**	5.4	**143 ^ab^**	4.5	**148 ^ab^**	5.3	**146 ^ab^**	11.1	**162 ^a^**	14.1
Day 16	**89 ^b^**	4.8	**93 ^ab^**	8.1	**102 ^ab^**	2.6	**101 ^ab^**	2.8	**104 ^ab^**	4.5	**112 ^a^**	13.9	**110 ^ab^**	12.5	**108 ^ab^**	3.0
Biomass gain ^1^ /rep, fresh	**7.94 ^c^**	0.480	**8.23 ^bc^**	0.435	**9.12 ^abc^**	0.463	**9.06 ^abc^**	0.469	**9.33 ^ab^**	0.274	**8.21 ^bc^**	0.441	**9.08 ^abc^**	0.598	**9.60 ^a^**	0.131
Biomass gain ^1^ g/rep, DM	**2.56 ^ab^**	0.147	**2.43 ^ab^**	0.196	**2.67 ^ab^**	0.113	**2.55 ^ab^**	0.232	**2.67 ^ab^**	0.059	**2.34 ^b^**	0.150	**2.87 ^a^**	0.233	**2.60 ^ab^**	0.214
Fat gain, g/rep	**0.63 ^a^**	0.066	**0.54 ^ab^**	0.026	**0.67 ^a^**	0.096	**0.34 ^c^**	0.064	**0.43 ^bc^**	0.21	**0.42 ^bc^**	0.045	**0.40 ^bc^**	0.040	**0.32 ^c^**	0.005
CP gain, g/rep	**1.14 ^ab^**	0.065	**1.13 ^ab^**	0.092	**1.20 ^ab^**	0.051	**1.12 ^ab^**	0.102	**1.16 ^ab^**	0.026	**1.00 ^b^**	0.064	**1.27 ^a^**	0.103	**1.15 ^ab^**	0.095
AA gain, g/rep	**0.77 ^ab^**	0.044	**0.75 ^ab^**	0.061	**0.82 ^ab^**	0.035	**0.77 ^ab^**	0.069	**0.75 ^ab^**	0.017	**0.68 ^b^**	0.044	**0.84 ^a^**	0.069	**0.79 ^ab^**	0.065
Conversion efficiency ^1^ g/g	**0.21 ^a^**	0.011	**0.18 ^b^**	0.006	**0.19 ^ab^**	0.006	**0.21 ^ab^**	0.016	**0.20 ^ab^**	0.011	**0.18 ^ab^**	0.010	**0.21 ^ab^**	0.009	**0.20 ^ab^**	0.008
Substrate per gain, g/g	**9.77 ^ab^**	0.578	**10.33 ^ab^**	0.835	**9.39 ^ab^**	0.396	**9.85 ^ab^**	0.851	**9.36 ^ab^**	0.209	**10.70 ^a^**	0.662	**8.74 ^b^**	0.723	**9.66 ^ab^**	0.828

^1^ Biomass gain, g: final biomass, g—initial biomass, g; Conversion efficiency, g biomass/(g substrate-g frass), on DM basis: biomass, mg DM/(Substrate, g DM—Frass, g DM), Different superscripts within a row indicate significant differences: ^a–c^ with *p* < 0.05, Tukey test; treatment means in bold; SD: standard deviation.

**Table 3:** Nitrogen balance and balance of sum of amino acids of BSFL grown for 16 days on substrates differing in their amino acid profiles.

	Trt 1 PC	SD	Trt 2 -Met	SD	Trt 3 -Cys	SD	Trt 4 -Lys	SD	Trt 5 -Arg	SD	Trt 6 -Phe	SD	Trt 7 -His	SD	Trt 8 +Met	SD
N balance, % of substrate																
Biomass	**36.5 ^ab^**	2.09	**36.8 ^ab^**	2.97	**39.4 ^ab^**	1.67	**38.0 ^ab^**	3.46	**39.9 ^ab^**	0.88	**33.1 ^b^**	2.11	**42.0 ^a^**	3.41	**36.2 ^ab^**	2.98
Frass	**54.1 ^abc^**	3.72	**52.6 ^abc^**	4.46	**49.2 ^abc^**	1.35	**56.7 ^ab^**	6.15	**46.9 ^bc^**	3.21	**57.7 ^a^**	1.00	**45.5 ^c^**	2.86	**48.7 ^abc^**	4.10
Loss	**9.3 ^ab^**	2.77	**10.6 ^ab^**	2.94	**11.4 ^ab^**	1.70	**5.3 ^b^**	4.69	**13.3 ^ab^**	3.12	**9.3 ^ab^**	2.39	**12.5 ^ab^**	1.94	**15.1 ^a^**	2.24
Sum of amino acids, % of substrate																
Biomass	**28.5**	1.63	**28.1**	2.27	**31.4**	1.33	**29.0**	2.63	**28.9**	0.64	**26.6**	1.70	**31.5**	2.56	**28.5**	2.35
Frass	**39.4 ^ab^**	2.71	**38.4 ^ab^**	3.26	**37.2 ^ab^**	1.02	**40.8 ^ab^**	4.42	**34.0 ^ab^**	2.33	**42.3 ^a^**	0.73	**34.1 ^b^**	2.14	**33.8 ^b^**	2.85
Loss	**32.0 ^abc^**	2.00	**33.5 ^abc^**	2.13	**31.4 ^bc^**	1.33	**30.2 ^c^**	3.36	**37.2 ^ab^**	2.26	**31.1 ^c^**	1.89	**34.4 ^abc^**	1.45	**37.7 ^a^**	1.52

Different superscripts within a row indicate significant differences: ^a–c^ with *p* < 0.05, Tukey test; treatment means in bold; SD: standard deviation.

**Table 4:** Average crude protein (CP) and lysine content of BSFL at harvest and respective amino acid profiles expressed in % of lysine ^1^.

	CP, % in DM	Lys, % in DM	Met	Cys	Thr	Arg	Ile	Leu	Val	His	Phe	Gly	Ser	Pro	Ala	Asp	Glu	Sum of AA: CP ^2^
Trt 1 PC	44.3	2.244	30	12	61	84	68	112	96	53	64	87	65	101	91	152	164	68
Trt 2 -Met	46.7	2.318	30	12	61	84	68	112	95	52	63	87	65	100	91	151	162	66
Trt 3 -Cys	45.2	2.290	29	12	62	84	68	111	96	53	63	88	66	101	95	151	166	68
Trt 4 -Lys	43.9	2.222	30	13	62	84	69	112	97	52	63	88	66	103	94	151	167	68
Trt 5 -Arg	43.3	2.018 ^3^	30	13	64	86	72	118	101	55	64	91	70	105	100	154	174	65
Trt 6 -Phe	42.8	2.117	30	13	63	86	70	115	97	53	64	88	67	101	95	152	171	67
Trt 7 -His	44.3	2.207	29	12	61	83	69	113	94	45	63	86	66	99	96	145	166	66
Trt 8 +Met	44.3	2.194	29	13	63	85	70	117	99	53	62	90	69	103	99	150	175	68

^1^ no statistics as only one BSFL sample per treatment was analysed ^2^ Sum of AA: sum of all analysed amino acids excluding tryptophan and tyrosine ^3^ grey-coloured cells indicate amino acids which may differ from the others within column.

**Table 5:** Amino acid retention efficiencies (in % of substrate) of BSFL grown for 16 days on substrates differing in their amino acid profile ^1^.

	Trt 1 PC	SD	Trt 2 -Met	SD	Trt 3 -Cys	SD	Trt 4 -Lys	SD	Trt 5 -Arg	SD	Trt 6 -Phe	SD	Trt 7 -His	SD	Trt 8 +Met	SD
Lysine	**40.0 ^b^**	2.29	**36.9 ^b^**	2.98	**41.8 ^b^**	1.77	**94.4 ^a^**	8.58	**35.8 ^b^**	0.79	**34.2 ^b^**	2.18	**42.2 ^b^**	3.43	**38.7 ^b^**	3.20
Methionine	**33.9 ^b^**	1.94	**80.7 ^a^**	6.52	**35.8 ^b^**	1.51	**32.1 ^b^**	2.91	**28.7 ^b^**	0.63	**29.5 ^b^**	1.89	**34.4 ^b^**	2.80	**19.5 ^c^**	1.61
Cysteine	**15.1 ^b^**	0.86	**14.7 ^b^**	1.18	**39.0 ^a^**	1.65	**16.3 ^b^**	1.48	**15.7 ^b^**	0.35	**14.1 ^b^**	0.91	**16.9 ^b^**	1.37	**16.7 ^b^**	1.37
Threonine	**31.3**	1.79	**29.8**	2.41	**33.9**	1.43	**30.9**	2.81	**31.6**	0.70	**28.8**	1.84	**34.4**	2.80	**32.0**	2.64
Arginine	**28.4 ^b^**	1.63	**26.7 ^b^**	2.16	**29.5 ^b^**	1.25	**27.7 ^b^**	2.52	**76.8 ^a^**	1.69	**25.5 ^b^**	1.63	**30.2 ^b^**	2.45	**27.3 ^b^**	2.25
Valine	**40.0 ^ab^**	2.29	**38.2 ^ab^**	3.09	**42.6 ^a^**	1.80	**38.8 ^ab^**	3.53	**37.8 ^ab^**	0.84	**34.5 ^b^**	2.21	**42.1 ^ab^**	3.42	**39.8 ^ab^**	3.27
Isoleucine	**34.5 ^ab^**	1.97	**33.8 ^ab^**	2.73	**37.8 ^a^**	1.60	**34.0 ^ab^**	3.09	**33.4 ^ab^**	0.74	**30.8 ^b^**	1.97	**37.7 ^a^**	3.06	**35.2 ^ab^**	2.90
Leucine	**27.8**	1.59	**27.0**	2.18	**30.1**	1.27	**27.0**	2.46	**26.4**	0.59	**25.0**	1.60	**30.4**	2.47	**28.9**	2.38
Phenylalanine	**25.8 ^b^**	1.48	**25.1 ^b^**	2.03	**27.7 ^b^**	1.17	**24.9 ^b^**	2.26	**24.6 ^b^**	0.54	**58.5 ^a^**	3.74	**28.4 ^b^**	2.30	**24.9 ^b^**	2.06
Histidine	**35.8 ^b^**	2.05	**34.6 ^b^**	2.80	**40.6 ^b^**	1.71	**36.8 ^b^**	3.35	**36.4 ^b^**	0.81	**32.4 ^b^**	2.07	**86.3 ^a^**	7.00	**36.8 ^b^**	3.04
Glycine	**36.5 ^ab^**	2.09	**36.9 ^ab^**	2.98	**40.4 ^a^**	1.71	**35.3 ^ab^**	3.21	**34.0 ^ab^**	0.75	**32.7 ^b^**	2.09	**40.6 ^a^**	3.29	**38.5 ^ab^**	3.18
Serine	**27.6**	1.57	**27.9**	2.25	**30.6**	1.29	**27.6**	2.51	**30.1**	0.66	**28.0**	1.78	**32.5**	2.64	**30.0**	2.48
Proline	**31.4 ^ab^**	1.79	**30.8 ^ab^**	2.49	**35.0 ^a^**	1.48	**31.2 ^ab^**	2.84	**29.9 ^ab^**	0.66	**27.9 ^b^**	1.78	**34.2 ^ab^**	2.78	**31.1 ^ab^**	2.56
Alanine	**37.4 ^ab^**	2.14	**36.5 ^ab^**	2.95	**42.7 ^a^**	1.80	**37.6 ^ab^**	3.41	**37.4 ^ab^**	0.82	**34.6 ^b^**	2.21	**42.2 ^a^**	3.42	**39.1 ^ab^**	3.23
Aspartate	**33.7**	1.93	**32.2**	2.60	**35.8**	1.52	**32.9**	2.99	**31.6**	0.70	**29.5**	1.88	**35.5**	2.87	**32.5**	2.67
Glutamate	**15.4**	0.88	**15.2**	1.23	**17.2**	0.73	**15.4**	1.39	**15.6**	0.34	**14.4**	0.92	**17.1**	1.39	**16.1**	1.33

^1^ grey-coloured cells indicate retention efficiencies of those amino acids which were reduced in substrates of the respective treatment; different superscripts within a row indicate significant differences: ^a–c^ with *p* < 0.05, Tukey test; treatments means in bold; SD: standard deviation.

**Table 6:** Losses of amino acids (% of substrate) of BSFL grown for 16 days on substrates differing in their amino acid profile.

	Trt 1 PC	SD	Trt 2 -Met	SD	Trt 3 -Cys	SD	Trt 4 -Lys	SD	Trt 5 -Arg	SD	Trt 6 -Phe	SD	Trt 7 -His	SD	Trt 8 +Met	SD
Lysine ^1^	**19.8 ^b^**	2.08	**27.8 ^ab^**	2.08	**23.3 ^ab^**	1.66	**−87.0 ^c^**	7.86	**34.3 ^a^**	2.01	**24.8 ^ab^**	2.34	**25.5 ^ab^**	2.11	**28.1 ^ab^**	1.71
Methionine ^1^	**28.2 ^c^**	1.93	**−66.6 ^d^**	4.85	**29.4 ^c^**	1.44	**32.8 ^c^**	2.91	**44.4 ^b^**	1.80	**33.0 ^c^**	2.03	**35.1 ^c^**	1.64	**61.5 ^a^**	1.89
Cysteine ^1^	**48.6 ^a^**	2.01	**46.6 ^ab^**	2.51	**−11.4 ^c^**	2.04	**40.2 ^b^**	3.91	**47.7 ^a^**	2.45	**46.5 ^ab^**	1.16	**49.7 ^a^**	1.24	**46.0 ^ab^**	2.11
Threonine	**15.6**	2.77	**17.8**	3.05	**15.1**	1.58	**13.0**	4.75	**20.3**	3.20	**15.4**	2.13	**17.7**	1.74	**19.9**	2.30
Arginine ^1^	**37.9 ^b^**	1.71	**43.3 ^ab^**	1.68	**40.8 ^ab^**	1.21	**39.9 ^ab^**	2.67	**−51.3 ^c^**	4.99	**40.7 ^ab^**	1.77	**42.8 ^ab^**	1.43	**45.7 ^a^**	2.26
Valine	**8.0 ^c^**	2.64	**13.1 ^bc^**	2.70	**9.4 ^c^**	1.77	**11.2 ^bc^**	4.10	**22.2 ^a^**	2.67	**14.8 ^bc^**	2.42	**16.6 ^abc^**	1.96	**17.6 ^ab^**	1.93
Isoleucine	**15.4 ^bc^**	2.56	**19.1 ^bc^**	2.63	**14.6 ^c^**	1.63	**17.7 ^bc^**	3.98	**27.2 ^a^**	2.62	**18.4 ^bc^**	2.20	**22.4 ^ab^**	1.74	**22.2 ^ab^**	1.93
Leucine	**30.1 ^b^**	2.16	**33.6 ^b^**	2.21	**31.1 ^b^**	1.31	**31.8 ^b^**	3.41	**40.3 ^a^**	2.22	**31.5 ^b^**	1.81	**34.9 ^ab^**	1.41	**34.2 ^b^**	1.68
Phenylalanine ^1^	**36.4 ^b^**	1.93	**39.4 ^ab^**	1.98	**37.4 ^b^**	1.19	**39.2 ^ab^**	2.97	**45.4 ^a^**	2.00	**−60.0 ^c^**	4.22	**39.9 ^ab^**	1.32	**42.9 ^ab^**	1.46
Histidine ^1^	**34.3 ^a^**	1.65	**36.4 ^a^**	1.86	**30.6 ^a^**	1.59	**31.8 ^a^**	2.81	**37.3 ^a^**	1.79	**36.5 ^a^**	2.17	**−50.5 ^b^**	4.34	**36.4 ^a^**	1.65
Glycine	**10.0 ^b^**	2.74	**9.3 ^b^**	3.01	**8.3 ^b^**	1.75	**12.5 ^b^**	4.31	**23.8 ^a^**	2.80	**12.4 ^b^**	2.35	**12.2 ^b^**	1.89	**13.2 ^b^**	2.19
Serine	**28.1**	2.29	**26.3**	2.63	**27.4**	1.36	**25.2**	3.96	**27.4**	2.82	**21.7**	2.03	**25.2**	1.60	**28.0**	1.95
Proline	**37.3 ^ab^**	1.62	**37.1 ^ab^**	1.82	**34.4 ^b^**	1.39	**33.8 ^b^**	2.90	**40.2 ^a^**	2.00	**38.1 ^ab^**	1.91	**35.7 ^ab^**	1.63	**38.3 ^ab^**	1.43
Alanine	**1.9 ^b^**	3.15	**4.5 ^ab^**	3.37	**0.4 ^b^**	1.88	**2.1 ^b^**	5.02	**1.5 ^b^**	3.14	**3.2 ^ab^**	2.51	**7.9 ^ab^**	1.98	**11.0 ^a^**	2.27
Aspartate	**20.5 ^c^**	2.33	**25.5 ^bc^**	2.34	**22.4 ^c^**	1.51	**22.6 ^c^**	3.65	**32.3 ^a^**	2.41	**23.4 ^bc^**	2.10	**26.5 ^abc^**	1.64	**29.2 ^ab^**	1.73
Glutamate	**59.4 ^ab^**	1.31	**59.5 ^ab^**	1.45	**58.4 ^b^**	0.78	**58.7 ^ab^**	2.18	**62.3 ^a^**	1.47	**59.0 ^ab^**	2.18	**61.1 ^ab^**	0.82	**61.7 ^ab^**	1.04

^1^ grey-coloured cells indicate losses of those amino acids which were reduced in substrates of the respective treatments; different superscripts within a row indicate significant differences: ^a–d^ with *p* < 0.05, Tukey test; treatment means in bold; SD: standard deviation.

## Data Availability

All data generated within this experiment are presented within this article.
